# Differential changes in the onset of spring across US National Wildlife Refuges and North American migratory bird flyways

**DOI:** 10.1371/journal.pone.0202495

**Published:** 2018-09-12

**Authors:** Eric K. Waller, Theresa M. Crimmins, Jessica J. Walker, Erin E. Posthumus, Jake F. Weltzin

**Affiliations:** 1 U.S. Geological Survey, Western Geographic Science Center, Menlo Park, California, United States of America; 2 National Coordinating Office, USA National Phenology Network, Tucson, Arizona, United States of America; 3 School of Natural Resources and the Environment, University of Arizona, Tucson, Arizona, United States of America; 4 U.S. Geological Survey, Western Geographic Science Center, Tucson, Arizona, United States of America; 5 U.S. Geological Survey, Tucson, Arizona, United States of America; University of Colorado, UNITED STATES

## Abstract

Warming temperatures associated with climate change can have indirect effects on migratory birds that rely on seasonally available food resources and habitats that vary across spatial and temporal scales. We used two heat-based indices of spring onset, the First Leaf Index (FLI) and the First Bloom Index (FBI), as proxies of habitat change for the period 1901 to 2012 at three spatial scales: the US National Wildlife Refuge System; the four major bird migratory flyways in North America; and the seasonal ranges (i.e., breeding and non-breeding grounds) of two migratory bird species, Blue-winged Warbler (*Vermivora cyanoptera*) and Whooping Crane (*Grus americana*). Our results show that relative to the historical range of variability, the onset of spring is now earlier in 76% of all wildlife refuges and extremely early (i.e., exceeding 95% of historical conditions) in 49% of refuges. In all flyways but the Pacific, the rate of spring advance is generally greater at higher latitudes than at lower latitudes. This differential rate of advance in spring onset is most pronounced in the Atlantic flyway, presumably because of a “warming hole” in the southeastern US. Both FLI and FBI have advanced markedly in the breeding ranges–but not the non-breeding ranges–of the two selected bird species, albeit with considerable intra-range variation. Differences among species in terms of migratory patterns and the location and extent of seasonal habitats, as well as shifts in habitat conditions over time, may complicate predictions of the vulnerability of migratory birds to climate change effects. This study provides insight into how differential shifts in the phenology of disparate but linked habitats could inform local- to landscape-scale management strategies for the conservation of migratory bird populations.

## Introduction

Warming temperatures associated with climate change are having measurable effects on diverse plant and animal species. Plants have demonstrated widespread advances in the timing of spring phenology [1,2], as well as shifts in distribution poleward and toward higher elevations [3,4]. Birds have similarly exhibited poleward habitat shifts [5–7], changes in abundance [8], and alterations in the timing of migratory and breeding behavior [9–13].

Long-distance migratory bird species, in particular, may be sensitive to changes in timing of seasonal transitions [14–16] given their reliance on ecological conditions at widely spaced wintering and breeding habitats [17]. The metabolic demands of extensive flight journeys mean that migratory and reproductive success hinge on the availability of sufficient food resources and optimal habitat conditions at stopover locations and upon arrival at breeding grounds [18,19]. Although many Northern Hemisphere species have adjusted the timing of their arrival at breeding areas [20,21], it is unclear whether shifts in migratory timing are able to keep pace with alterations in plant phenology and food resource availability across broadly distributed habitats [13,22–25]. Migratory bird species display remarkable inconsistency in their short-term phenotypic adaptations to climatic warming. Sources of variation include the relative degree of reliance on endogenous or environmental cues to initiate migration [21], environmental and climatic conditions en route [26,27], and intra-species heterogeneity in migration arrival timing [20,28].

The association of spring green-up with bird reproductive events [25] and the abundance of insects, a key food source [29,13,30] makes shifts in timing of spring onset a useful surrogate for climate change impacts relevant to migratory species. The evaluation of seasonality effects is complicated by the spatial heterogeneity of warming rates at regional scales [3] and the specificity of cues that trigger plant growth in individual species [31]. The combined effects contribute to a mosaic of seasonal patterns within and across ecological systems and migratory flyways (e.g., [13]). In the continental United States, the biological onset of spring is generally advancing, but the magnitude and even the direction of shifts are inconsistent across space and time [32,33]. For instance, depending on the time frame of reference, areas of the Southeast and interior Northwest are experiencing delayed onsets of spring [34]. Future climate projections point to a more rapid advance in spring onset at higher latitudes, which could reduce differences in spring onset dates between low and high latitudes [35,36]. The convergence of spring onset across latitudes may affect habitat conditions for migratory species such as birds ([37] but see [38]).

Spatiotemporal complexity in spring onset poses a challenge for agencies charged with managing or conserving migratory species, particularly given projections of rapid and spatially differentiated changes in climate at national to continental scales [36]. In the US, the federal Fish and Wildlife Service (USFWS) administers the National Wildlife Refuge System (NWRS), which encompasses a network of public lands reserved for wildlife conservation and management. The conservation of migratory birds is a central theme of the NWRS; more than 200 refuges were established specifically to provide breeding or wintering habitat [39]. In 1997, the NWRS recast management priorities to expand the scope of conservation beyond refuge boundaries to consider broader landscapes [40,41] and to establish a basic framework to address the effects of changing climate conditions [42].

Here, we evaluated how the timing of spring onset within the US and North America has changed over the past century at spatial scales relevant to bird species that follow migratory pathways across the continent to access seasonal habitats. We used biological onset of spring as an indicator of environmental change because of its potential direct and indirect effects on habitats and food resources accessed by migratory birds. We determined spring onset on the basis of the Extended Spring Indices (SI-x), which are models that simulate the biological start of spring based primarily on antecedent temperature conditions [33,43]. These indices have been used to demonstrate differential changes in the onset of spring across a variety of spatial extents, including the US National Park System [44], US regions [32], the continental US [33,45], North America [34], Europe [46], and China [47].

Our objectives for this study were to investigate changes in spring onset at multiple spatial scales relevant to resource management and migratory birds. First, we evaluated how relatively recent (1983–2012) timing of spring onset compares to historical (1901–2012) timing of spring onset for 496 individual refuges across the NWRS. This analysis provides a localized perspective of absolute changes and trends relative to the historical range of variability within any given refuge, as well as a broader overview of patterns of change across the NWRS. Second, to examine seasonal change dynamics along the entirety of migratory flight routes, we quantified rates of change in spring onset across latitudinal gradients within each of the four major migratory bird flyways of North America. Finally, to demonstrate how differential rates of advance in spring onset could affect migratory bird species, we conducted these analyses within the breeding and non-breeding ranges of two migratory bird species, Blue-winged Warbler (*Vermivora cyanoptera*) and Whooping Crane (*Grus americana*), which have different migratory patterns, ecological requirements, and population status.

## Materials and methods

### First leaf and first bloom indices of spring onset

We relied on two SI-x metrics of spring onset: First Leaf Index (FLI) and First Bloom Index (FBI) [33,43]. The indices use models of first leaf and first bloom in a cloned lilac cultivar (*Syringa x chinensis* 'Red Rothomagensis') and two cloned honeysuckle cultivars (*Lonicera tatarica* 'Arnold Red' and *L*. *korolkowii* 'Zabelii'). These heat-sum accumulation threshold models have been used to document changes in recurrent seasonal plant and animal activity across the continental US and North America [34]. The FLI represents the earliest spring-season activity in plants, as characterized by leaf-out in early-season shrubs, and is a function of accumulated heat and synoptic events after January 1. The FBI, which occurs later in the season, represents flowering of shrubs and leaf-out of deciduous trees, and is driven mainly by additional heat accumulated after the FLI threshold is met [48]. Historical annual maps and real-time and short-term forecasts of these indices are generated and maintained by the USA National Phenology Network (USA-NPN). The USA-NPN collects, stores, and shares phenology data and information products to advance science and support decision-making.

We obtained annual gridded maps of FLI and FBI in NetCDF format from the USA-NPN [49]. The indices were calculated using the Berkeley Earth daily minimum and maximum surface temperature data products, and span the period 1880 to 2013 over the region 180° to 0° W, 0° to 90° N. Values within each 1° latitude-square grid cell represent the day of year (DOY) on which the requirements for FLI and FBI were satisfied.

Following procedures outlined in [44], we generated gridded GeoTIFFs of 10-, 20-, and 30-year right-aligned moving window means and standard deviations for each index across the region of interest. For example, 10-yr moving window values were calculated for 125 periods (1880–1889, 1881–1890, 1882–1891, …, 2004–2013). The moving windows were designed to smooth potentially noisy annual data at temporal scales relevant to resource management strategy time frames (typically 10–20 years) and major climatic cycles such as the North Atlantic Oscillation (typically 10, 20, or 30 years) [50]. All analyses were performed using R [51] scripts (S1 Code).

### Onset of spring within the US National Wildlife Refuge System over the last century

To evaluate spatial and temporal patterns of FLI and FBI across the NWRS, we used the most recent digital coverage of National Wildlife Refuges [52]. We added a 30-km buffer around each unit and dissolved overlapping buffers into single polygons. We then merged records for individual refuges with multiple discrete polygons so that each refuge was represented by a single record in our data table, for a total of 512 unique refuge records. FLI and FBI data were not available for sixteen refuges (generally islands and atolls well off the North American continent), leaving 496 refuges for subsequent analysis.

For each refuge, we calculated annual FLI and FBI for the period 1901–2012. We restricted the range to 2012 to facilitate direct comparisons with the results of other studies (e.g., [44]). Following procedures and supplied code in [44] for all subsequent calculations in this section, we generated 10-, 20-, and 30-year moving windows for FLI and FBI separately for each refuge. Generally, for each index at each refuge, we determined area-weighted mean; recent timing and variability (based on averaging the most recent moving window means and standard deviations); recent change in timing relative to historical range of variation (HRV; i.e., distribution of moving window values); the sensitivity of the relative timing measure to moving window size; and temporal trends in timing, as described in the following paragraphs.

Area-weighted means for each index at each refuge were calculated by intersecting the buffered refuge boundaries with the raster layer of each index for each year. Recent timing and variability were calculated by taking the mean and standard deviation (SD) in annual FLI and FBI over each of the three most recent moving windows (2003–2012, 1993–2012, 1983–2012) and then averaging across the three moving window values for each index and SD.

For each index at each of the refuges, we computed the percentile of each most recent moving window average with respect to the HRV; we then averaged the three percentile values. We also computed the maximum difference in percentile (max Δ) among moving window means. These two values yield both a measure of recent timing of spring onset with respect to the range of historical conditions and an estimate of the measure’s sensitivity to trends in the past 30 years, respectively. Following the naming convention of [44], we hereafter refer to recent mean percentiles that are <5% as extremely early or advancing in timing of onset of spring; 5–25% as early; 25–75% as average; 75–95% as late or delaying in timing of onset of spring; and >95% as extremely late. Similarly, we categorize results for max Δ as low sensitivity to moving window size (<5%), moderate sensitivity (5–25%), and high sensitivity (>25%).

For each index at each of the refuges, we calculated temporal trends (d/decade) from the difference between the most recent moving window averages and their historic averages (e.g., for the 10-year approach, the average of all 10-year moving window averages from 1901–1910 to 1993–2002 was subtracted from the 2003–2012 average, and the result divided by 56 years, which is the difference between the midpoints of the most recent windows and the midpoints of the historical windows). We refer to these calculations as average moving window trends.

### Onset of spring within North American bird flyways over the last century

**Fig 1:**
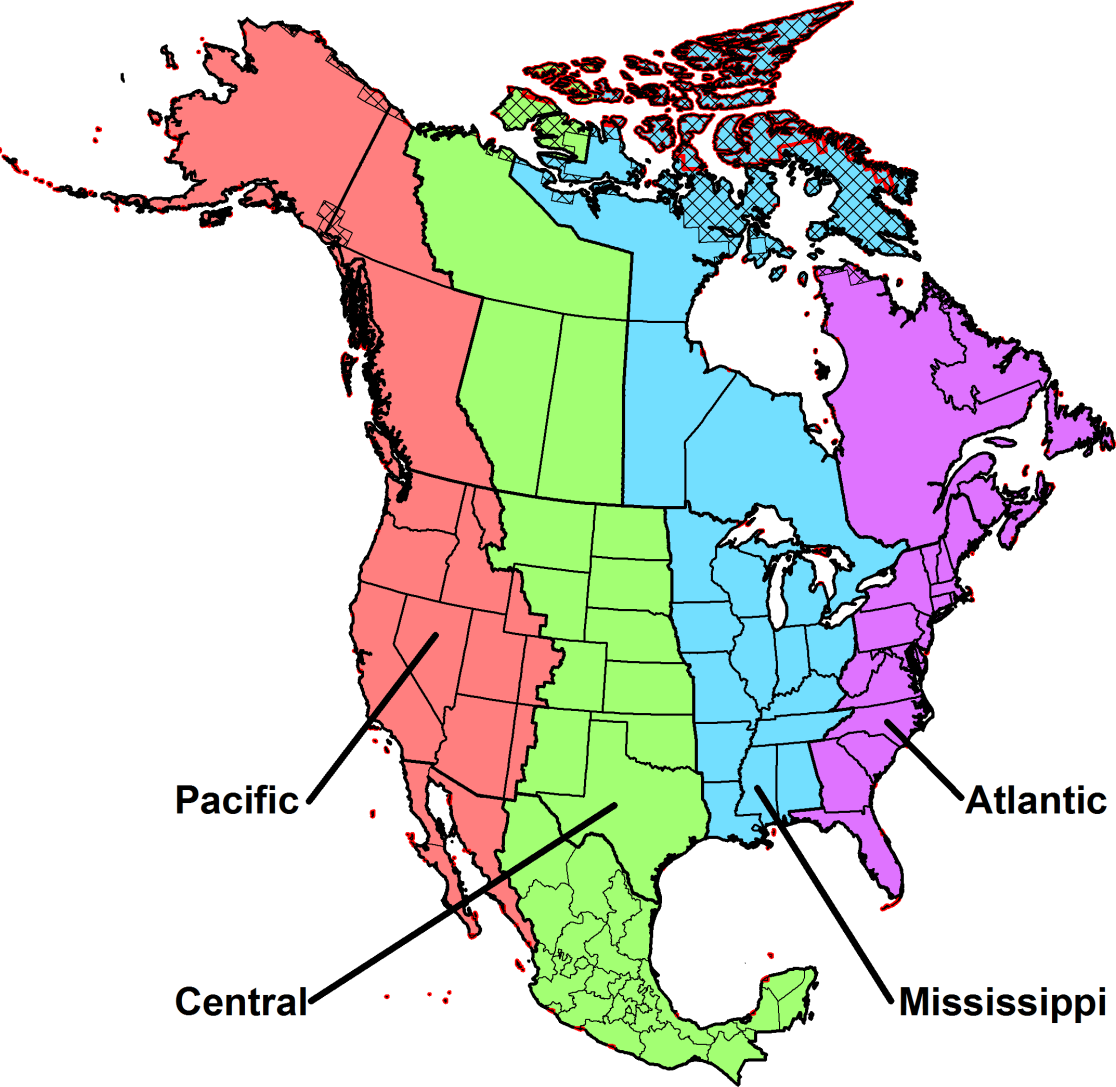
North American migratory bird flyway map. Regions shown as hatched or outlined in red had insufficient data for analyses of First Bloom Index (FBI) and First Leaf Index (FLI), respectively.

To evaluate whether spring onset has changed over time along the latitudinal extent of major North American migratory routes, we first created a continental-scale digital flyway map. The common system of four migratory flyways in North America was introduced in the mid-20^th^ century to provide a spatial framework for the management of migratory waterfowl [53]. Because a standard digital flyway map was not available, we replicated a non-digital USFWS flyway map [54] by merging a digital US flyway product [55] with digital boundaries of Canadian provinces [56] and Mexican states [57]. We manually defined boundaries to create the four distinct continental flyways: Pacific, Central, Mississippi and Atlantic (Fig 1). We restricted our analyses of FLI and FBI to the period 1920–2012 because of insufficient data at high latitudes early in the 20^th^ century, and we further excluded grid cells with two or more years of missing data.

We determined differential changes in spring onset by latitude. First, we used linear regression to determine the relationship between index DOY and latitude in each flyway—based on the 1-degree grid cell values—by year. For each index, flyway, and year, this calculation yielded a regression slope in days/1° latitude. We refer to this metric as the annual latitudinal slope of an index. Changes in latitudinal slopes over time indicate latitudinal asynchrony in changes in spring arrival. Changes in average flyway index value over time give context to changes in latitudinal slope (e.g., a change in latitudinal slope with no change in the average has different geographic implications than the same change with an earlier average). To determine temporal trends in average FLI and FBI for the four flyways, we calculated mean annual FLI and FBI for all grid cells pooled within each flyway, and then determined linear relationships between each index and year for each flyway. Similarly, for each flyway, we calculated temporal trends for the latitudinal slope of each index, by regressing the annual latitudinal slope values against year. This regression yielded a rate of change in advance in the latitudinal slope of FLI or FBI that we report in days/10° latitude/decade.

### Onset of spring in breeding and non-breeding ranges of two migratory bird species

To address how seasonal habitats of individual migratory bird species might be affected by different rates of spring advance along latitudinal gradients, we evaluated trends in spring onset within and between the breeding and non-breeding ranges of *Vermivora cyanoptera* (Blue-winged Warbler) and *Grus americana* (Whooping Crane) for the period 1901–2012. The two species are intended as demonstrations of the utility of the data for determining potential impacts on bird populations.

*V*. *cyanoptera* is a neotropical migrant with a non-breeding range in areas of the Caribbean, Central America, and Mexico, and an expansive breeding range in the northeastern U.S. and portions of southeastern Canada [58]. Breeding, wintering, and transitional habitats of *V*. *cyanoptera* fall within the Atlantic, Mississippi, and Central flyways. This species has demonstrated earlier arrival at breeding grounds, though the shift has lagged behind the advance in vegetation green up [13]. The critically endangered *G*. *americana* [59] has a comparatively limited distribution: the migratory population breeds in a small protected wetland in northeastern Alberta and south-central Northwest Territories of Canada (Wood Buffalo National Park), and winters in a similarly constrained wetland (Aransas National Wildlife Refuge) near Corpus Christi, Texas [58]. Breeding, wintering, and transitional habitats of *G*. *americana* are in the Central flyway. There is evidence that the migration corridor in the US Great Plains region has narrowed and shifted eastward over the past few decades, possibly in response to habitat availability [60], but spring and fall migration timing was invariant over a 57-year period of observation (1943–1999) [61].

For each species we evaluated changes in the onset of spring using FLI and FBI for breeding and non-breeding ranges. Using the methodology employed for the refuge analysis as described above, we calculated moving window mean and standard deviation for the 1901–2012 index data for each of the one-degree grid cells within the breeding and non-breeding ranges. We used the statistical distributions of the moving window means to estimate the HRV for each grid cell and determined the percentiles for the most recent moving windows. We averaged the three final moving window percentiles and calculated the maximum difference (max Δ) among percentiles.

We evaluated temporal trends in spring onset for the breeding and non-breeding ranges for each species by calculating linear regressions between the average annual index values pooled within range against year (1901–2012) for each combination of index, range, and species.

## Results

### Changes in onset of spring within US wildlife refuges over the last century

**Fig 2:**
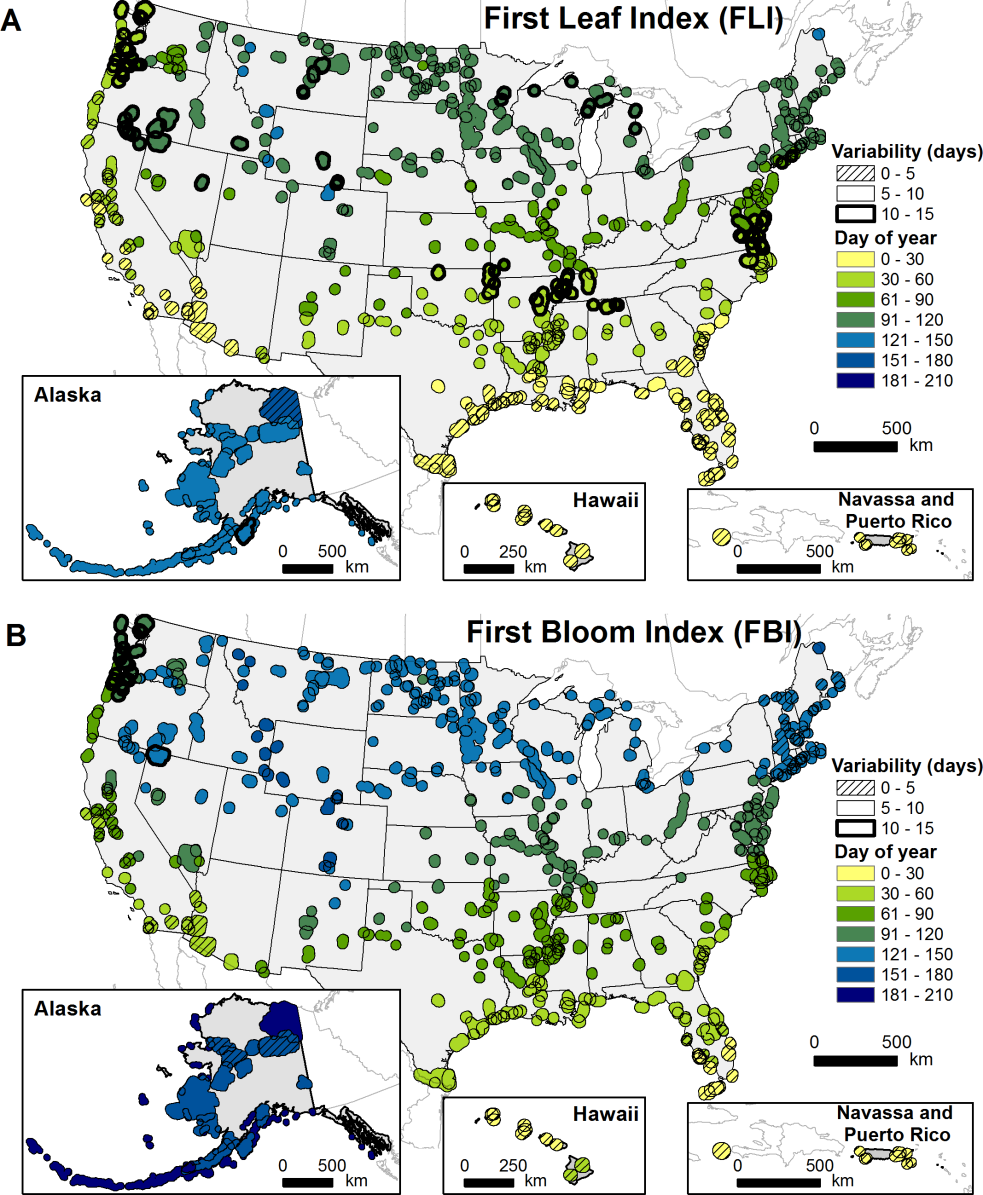
Recent timing of spring onset in US National Wildlife Refuges. Day of year and variability (i.e., standard deviation) calculated from the averages of the three most recent moving window values (1983–2012, 1993–2012, 2003–2012) for (A) First Leaf Index and (B) First Bloom Index.

Mean FLI and FBI exhibited clear latitudinal patterns, both occurring earliest within southern refuges and generally later in more northern refuges (Fig 2). Refuges located at higher latitudes, higher elevations, and within the continental interior generally exhibited later spring onset than refuges at lower latitudes, lower elevations, or within coastal regions. Across all refuges, FBI lagged FLI by approximately one month (DOY mean±SD; FLI: 68.1±37.0, FBI: 99.2±40.8). Refuges in the Caribbean and Hawaiian Islands and in southern Florida exhibited shorter durations between the two indices (<15 days), and refuges along the western coasts of Washington and Oregon exhibited longer durations between the two indices (>50 days).

**Fig 3:**
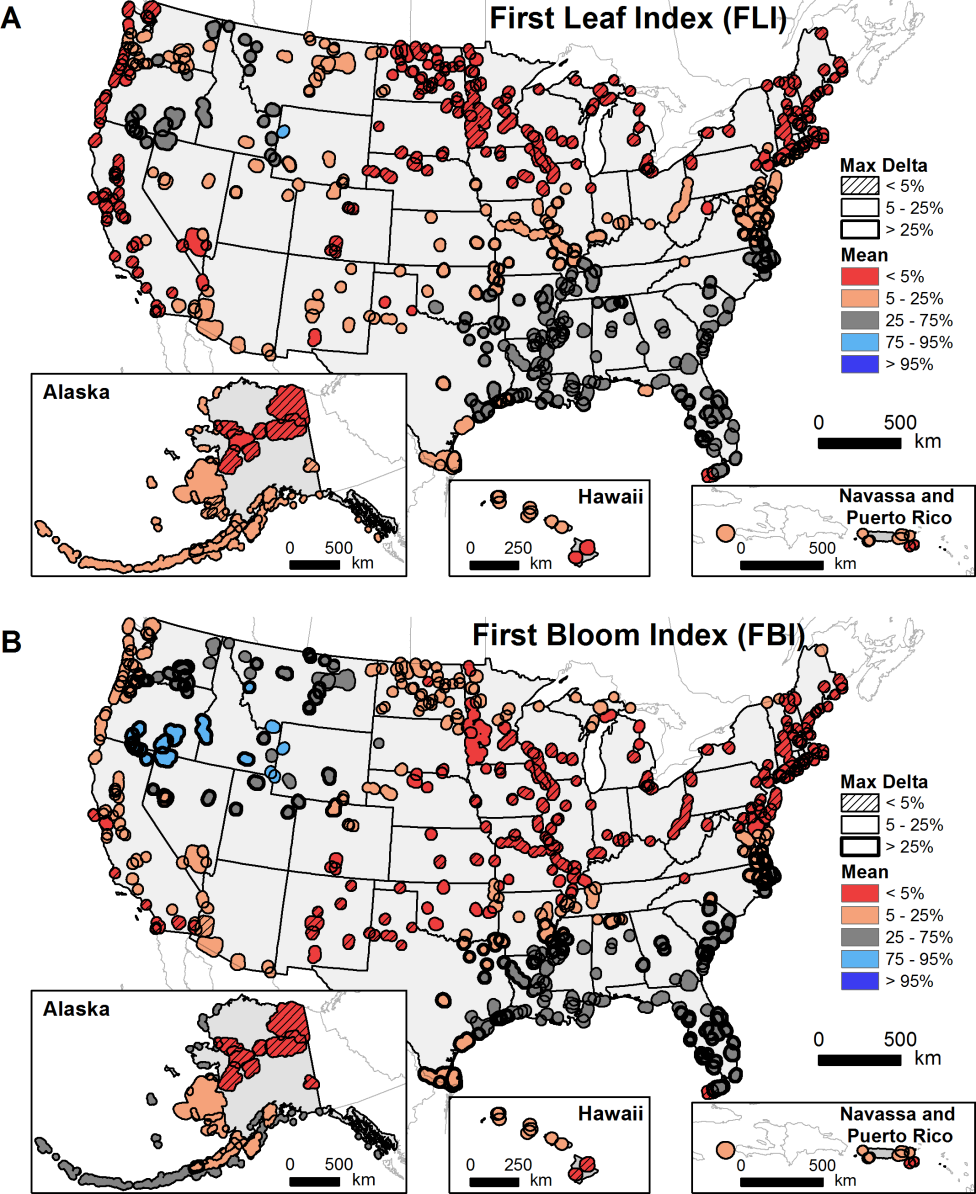
Recent timing of spring onset in US National Wildlife Refuges relative to the historical range of variability. Shown are the mean of the percentiles—relative to the 1901–2012 range of variability—of the most recent index moving window means (1983–2012, 1993–2012, 2003–2012); and the maximum difference between index moving window percentiles (“max delta”) for (A) First Leaf Index and (B) First Bloom Index.

The majority of refuges are experiencing earlier onsets of spring than in the early 20^th^ century (Fig 3). Three-hundred thirty-eight (68%) refuges exhibited early or extremely early FLI relative to the historical range of variability (HRV); similarly, 68% exhibited early or extremely early FBI (Table 1). Three percent of the refuges, roughly grouped in the southern portion of the interior Northwest, experienced delays in FBI. Only one refuge—the National Elk Refuge in Wyoming—had a delay in FLI. No refuges exhibited extreme delay in spring onset relative to HRV. Reponses for individual refuges are in S1 Table.

**Table 1:** Categorization of recent spring onset indices relative to historical range of variability for individual national wildlife refuges.

Percentile range	Change category	Number of refuges (%) in each range	
		FLI	FBI
0–5	Extremely early	175 (35%)	150 (30%)
5–25	Early	163 (33%)	187 (38%)
25–75	Normal	157 (32%)	142 (29%)
75–95	Late	1 (<1%)	17 (3%)
95–100	Extremely late	0	0

FLI, First Leaf Index; FBI, First Bloom Index.

**Fig 4:**
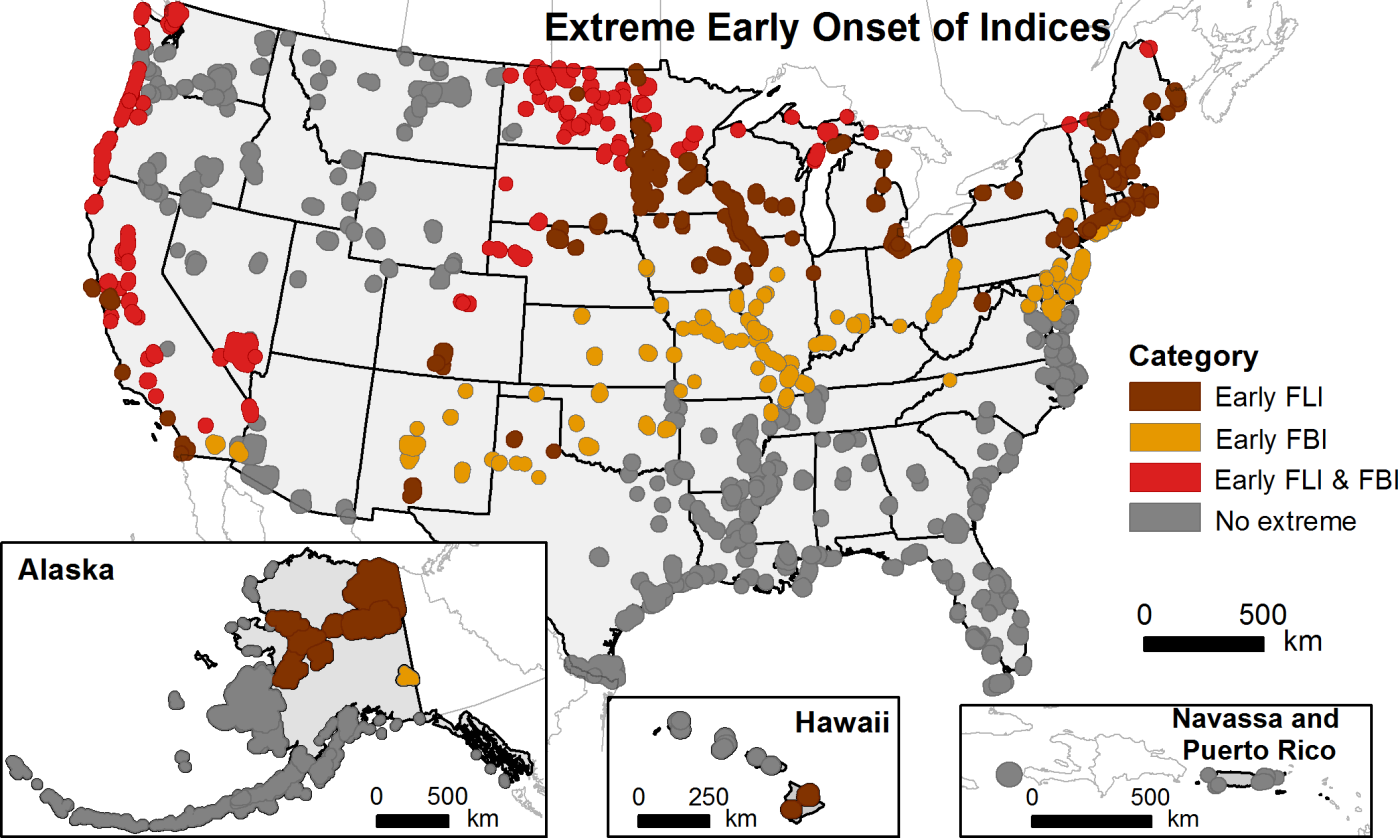
US National Wildlife Refuges with extremely early recent spring onset relative to the historical range of variability. Shown is the combination of selected data from Fig 3A and 3B; extremely early recent spring onset is defined as earlier than 95% of historical (1901–2012) First Leaf Index (FLI) or First Bloom Index (FBI) values.

Two-hundred forty-one (49%) refuges exhibited extremely early spring for either FLI, FBI, or both indices relative to HRV (Fig 4). Refuges along the Pacific coast, in the Mojave Desert, the northern Great Plains, and the upper Midwest exhibited both extremely early FLI and extremely early FBI. Refuges in the Northeast and Midwest, portions of the central and southern Great Plains, the Big Island of Hawaii, and in northern Alaska exhibited primarily extremely early FLI. Refuges in the middle latitudes exhibited mainly extremely early FBI. Across much of the Southeast and the interior Northwest, refuges exhibited average FLI and FBI relative to HRV.

A minority of refuges exhibited high sensitivity to moving window size: 113 (23%) and 121 (24%) refuges returned high (>25%) max Δ values for FLI and FBI, respectively. The majority of refuges with high max Δ values were in the Southeast and interior Northwest, where FLI and FBI were generally average relative to HRV (Fig 3). In contrast, the majority of refuges with extremely early FLI and/or FBI values relative to HRV showed low sensitivity (max Δ <5%) to moving window size.

For most refuges, trends in FLI and FBI were negative, indicating that recent onsets of spring are earlier than in the past (S1 Fig). Eighty-four percent of refuges exhibited a negative slope for FLI, and 84% of refuges exhibited a negative slope for FBI. Changes in FLI were as high as 3 d/decade, and changes in FBI were up to 2 d/decade (S1 Table).

### Changes in onset of spring within North American bird flyways over the last century

**Fig 5:**
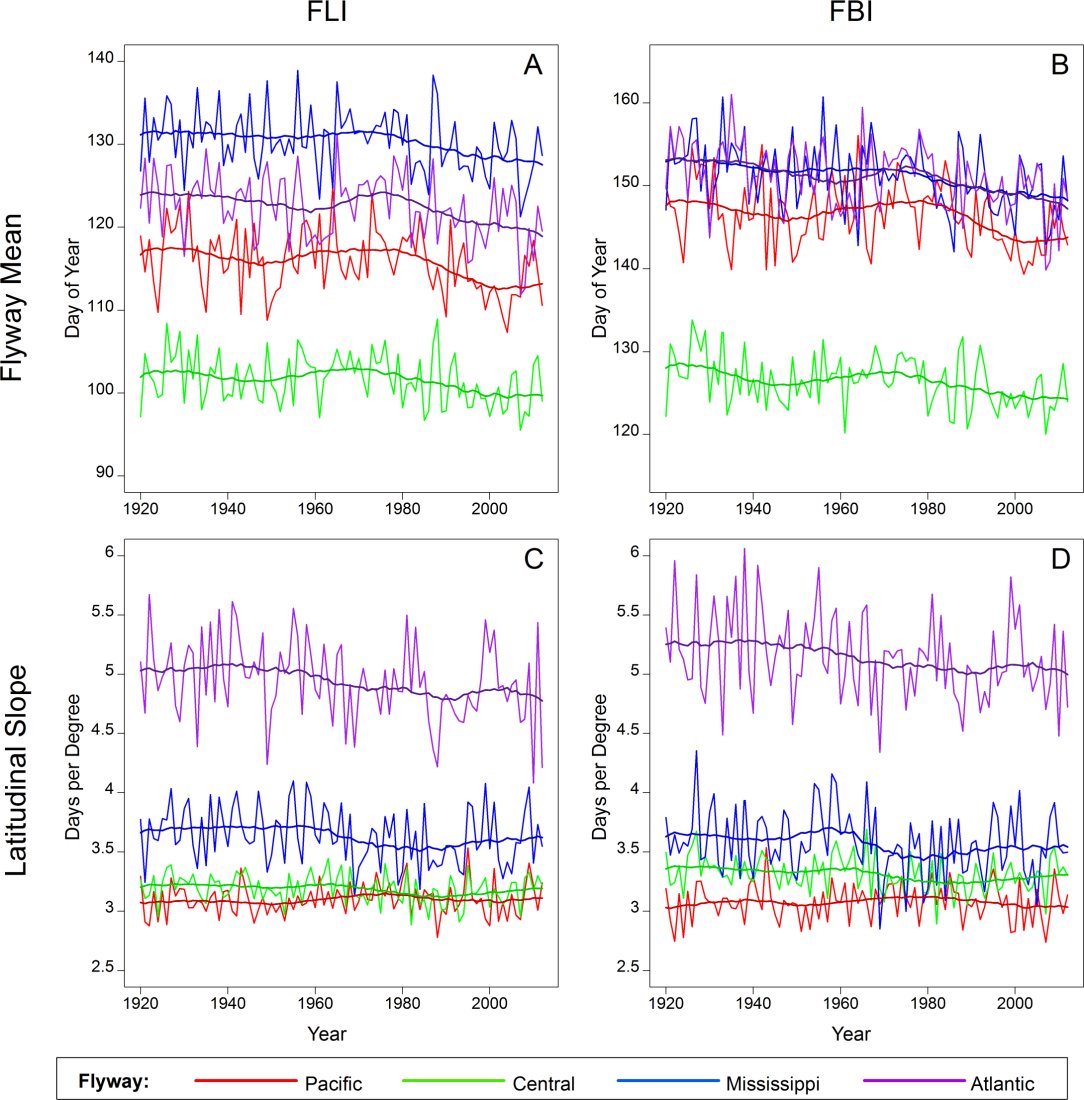
Spring onset and latitudinal slope of spring onset within migratory bird flyways. Shown are mean annual (A) First Leaf Index (FLI) and (B) First Bloom Index (FBI) and the annual slope between (C) FLI and latitude (d/1° latitude) and (D) FBI and latitude (d/1° latitude) for 1920–2012. Bold lines represent the average of the three centered moving window means (10-year, 20-year, 30-year).

All flyways exhibited significant trends towards earlier onset of spring as indicated by both FLI and FBI, though trends varied among the flyways (Fig 5A and 5B; Table 2). Over the period of record, FLI advanced between 0.27 d/decade (in the Central flyway) and 0.47 d/decade (in the Atlantic flyway). Similarly, FBI advanced between 0.37 (Central) and 0.56 d/decade (Atlantic).

**Table 2:** Trends in spring onset and latitudinal slope of spring onset by North American migratory flyway, 1920–2012.

Flyway	Metric	Trend (β) (d/decade)	*p*-value	Trend (β) in latitudinal slope (d/10° latitude/decade)	*p*-value^a^
Pacific	FLI	-0.441	0.003**	0.046	0.38
Central	FLI	-0.273	0.02*	-0.087	**0.07+**
Mississippi	FLI	-0.394	0.004**	-0.186	0.04*
Atlantic	FLI	-0.468	0.001**	-0.362	0.005**
Pacific	FBI	-0.409	0.006**	0.012	0.84
Central	FBI	-0.370	0.002**	-0.133	0.02*
Mississippi	FBI	-0.517	0.0005**	-0.189	**0.08+**
Atlantic	FBI	-0.562	0.0001**	-0.358	0.01**

FLI, First Leaf Index; FBI, First Bloom Index.

^*a*^ significance levels:

+, p ≤ .10

*, p ≤ .05

**, p ≤ .01.

Bolded values are significant.

The trend in the latitudinal slope for both FLI and FBI over the period of record was negative for the Central, Mississippi and Atlantic flyways (Fig 5C and 5D; Table 2), indicating a greater advance in onset of spring at northern latitudes than in southern latitudes. Differences in the rates of spring advance by latitude ranged from -0.36 d/10° latitude/decade for both FLI and FBI across the Atlantic flyway, to essentially no trend across the Pacific flyway (Table 2).

### Changes in onset of spring within and between breeding and non-breeding ranges of two migratory bird species

**Fig 6:**
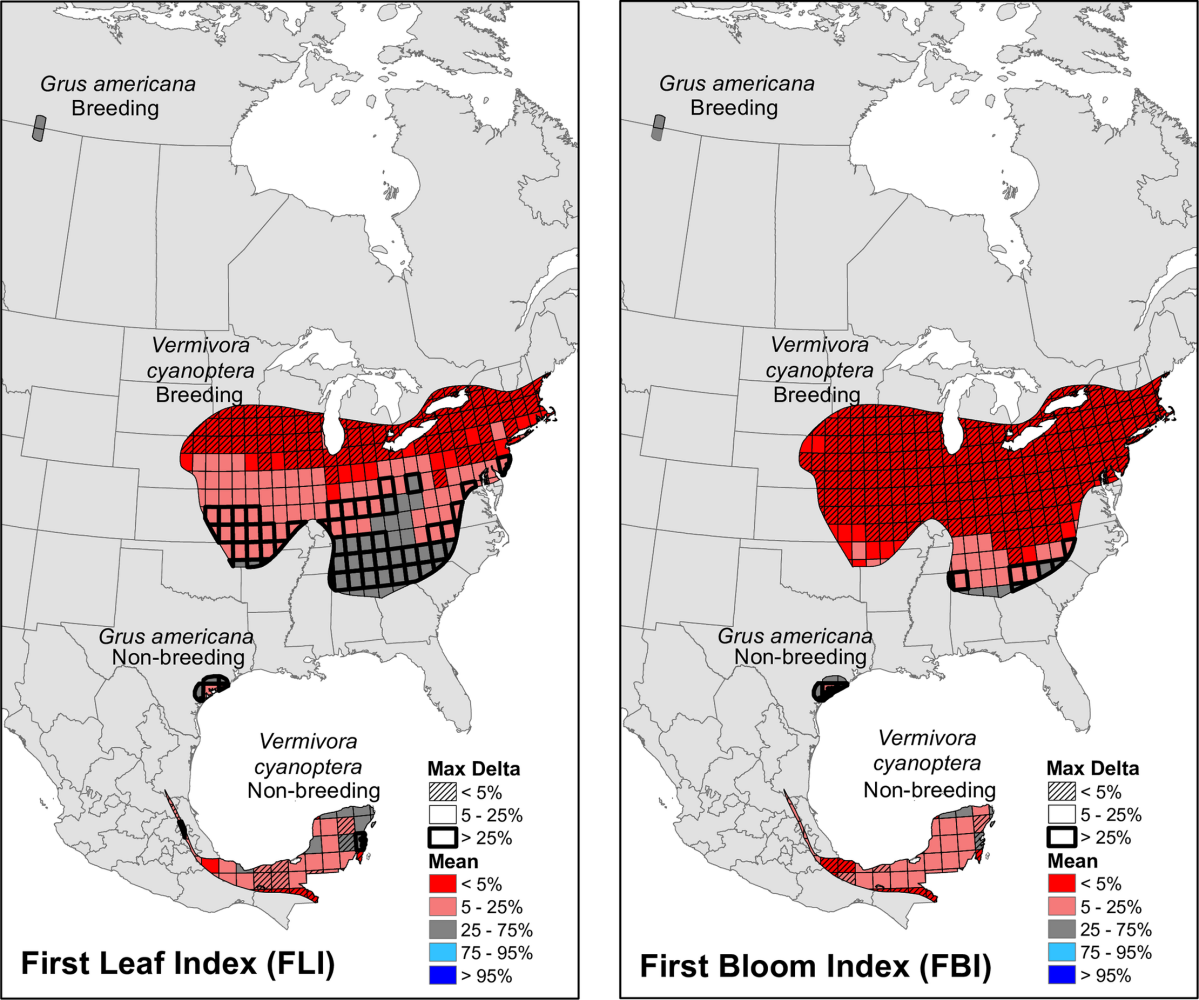
Recent timing of spring onset within seasonal habitats of *Vermivora cyanoptera* and *Grus americana* relative to the historical range of variability. Shown are the average of the percentiles—relative to the 1901–2012 range of variability—of the most recent index moving window means (1983–2012, 1993–2012, 2003–2012); and the maximum difference between index moving window percentiles (“max delta”) for (A) First Leaf Index (FLI) and (B) First Bloom Index (FBI). Grid cells represent the 1° resolution of analysis within the breeding and non-breeding ranges of each bird species.

Large portions of *V*. *cyanoptera* breeding and non-breeding ranges exhibited early springs as indicated by recent mean percentile values relative to HRV for both FLI and FBI (Fig 6). The majority of the non-breeding range shifted towards earlier spring for both FLI and FBI. Changes in spring onset in the breeding range differed between the two indices. FLI across the breeding range was normal, early, or extremely early in about-equal proportions, with the greatest extremes along the northern tier. In contrast, FBI was extremely early across the majority of the breeding range, with less change along the southern edge. Similar to individual refuges, grid cells with the greatest change in spring onset tended to exhibit the least sensitivity to moving window size (max Δ <5%). In contrast to *V*. *cyanoptera*, the majority of grid cells in both the breeding and non-breeding ranges of *G*. *americana* showed little change in timing of spring onset relative to HRV over the period of record (1901–2012).

**Fig 7:**
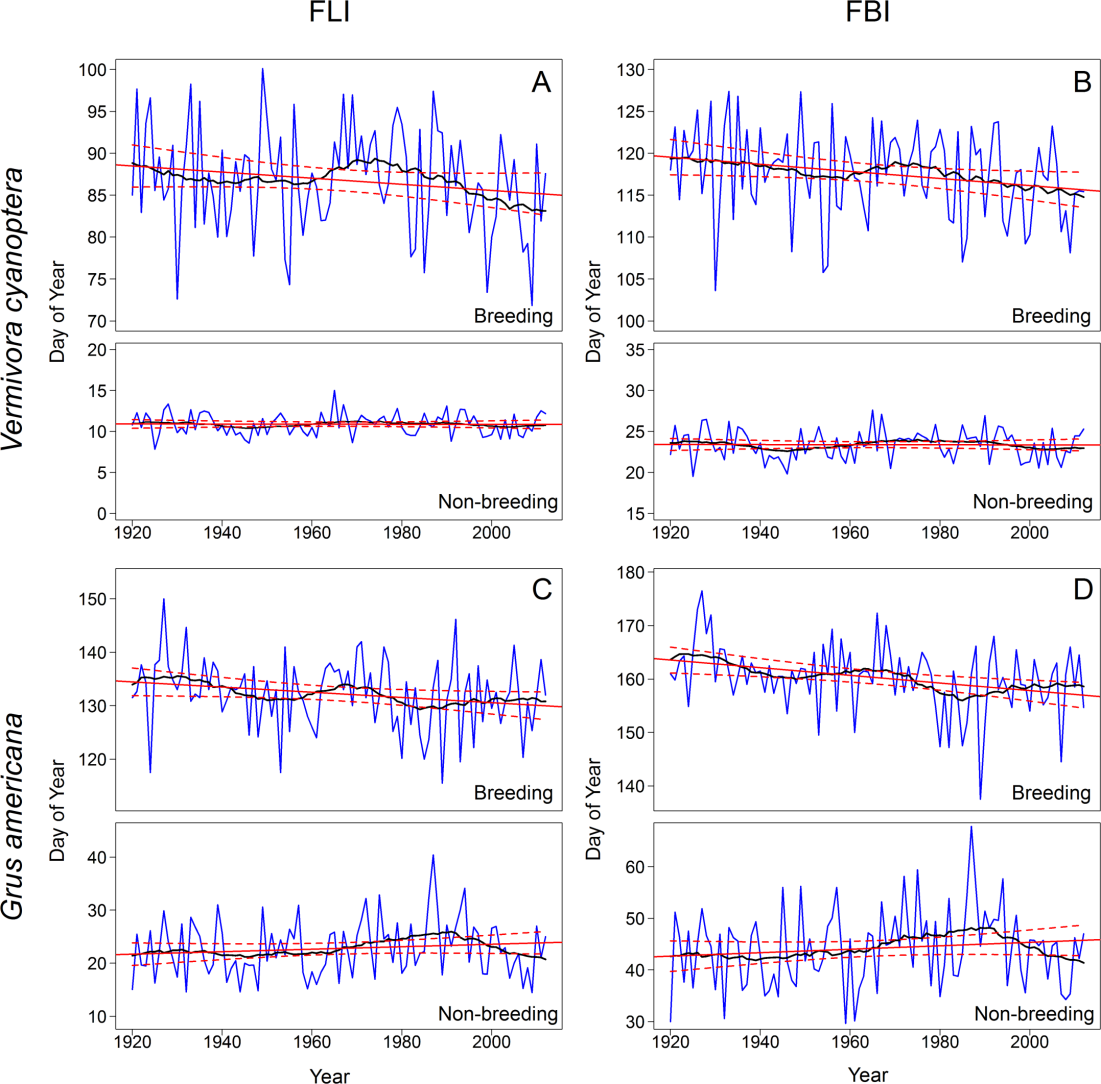
Spring onset values and trends for *Vermivora cyanoptera* and *Grus americana* seasonal habitats. Annual mean (A) First Leaf Index (FLI) and (B) First Bloom Index (FBI) for *V*. *cyanoptera* breeding and non-breeding ranges, and annual mean (C) FLI and (D) FBI for *G*. *americana* breeding and non-breeding ranges. Black lines represent the average of the three centered moving window means (10-year, 20-year, 30-year). Red lines represent fitted linear trends with 95% confidence intervals.

For both species, FLI and FBI averaged over the period of record were at least 2 months earlier in the non-breeding ranges than the breeding ranges. Over time, FBI in the *V*. *cyanoptera* breeding range advanced significantly on the order of 0.42 d/decade, whereas this index did not change significantly over time in the non-breeding range (Fig 7; Table 3). FLI advanced at a similar rate (0.36 d/decade) in the breeding range, but with greater interannual variability so the trend was not significant. FLI did not change significantly in the non-breeding range. FLI and FBI in the breeding range of *G*. *americana* advanced significantly by 0.49 d/decade and 0.72 d/decade, respectively, whereas these indices did not change significantly over time in the non-breeding ranges.

**Table 3:** Trends in spring onset in *Vermivora cyanoptera* and *Grus americana* seasonal ranges, 1901–2012. Annual means of First Leaf Index (FLI) and First Bloom Index (FBI) in each range are in Fig 7A and 7B for *V*. *cyanoptera* and in Fig 7C and 7D for *G*. *americana*.

Species	Metric	Range	Trend (β) (d/decade)	*p*-value^a^
*V*. *cyanoptera*	FLI	Breeding	-0.36	0.13
*V*. *cyanoptera*	FLI	Non-breeding	-0.007	0.90
*V*. *cyanoptera*	FBI	Breeding	**-0.42**	0.037*
*V*. *cyanoptera*	FBI	Non-breeding	-0.005	0.94
*G*. *americana*	FLI	Breeding	**-0.49**	0.049*
*G*. *americana*	FLI	Non-breeding	0.23	0.25
*G*. *americana*	FBI	Breeding	**-0.72**	0.002**
*G*. *americana*	FBI	Non-breeding	0.33	0.24

FLI, First Leaf Index; FBI, First Bloom Index.

^*a*^ significance levels

*, p ≤ .05

**, p ≤ .01.

Bolded values are significant.

## Discussion

In this study, we used two indices of spring onset to evaluate changes in the start of spring at multiple spatial scales relevant to management of habitats for migratory birds in North America. We first examined how the onset of spring has changed at individual national wildlife refuges to provide relevant information and historical context to resource managers within the USFWS. We then evaluated changes in onset of spring within North American flyways, because migrating birds may be affected by spatial and temporal variation in seasonal habitat conditions beyond the boundaries of individual refuges or the refuge system. Finally, we examined spatial and temporal variation in spring onset within and between breeding and non-breeding ranges of two migratory bird species to elucidate how differential changes in seasonality between ranges might affect habitat conditions and migratory behavior. The results of our study are applicable to all mid-to-long-distance migratory species that access winter and breeding areas across North America. The processing and analysis techniques demonstrated here can be extended to other continents or spatial domains that include similar migratory corridors. In addition, the availability of long-term climatic data at a higher spatial resolution would allow for comparable analyses of species that migrate over shorter distances.

### Changes in spring onset within US national wildlife refuges over the last century

The onset of spring has advanced notably in the majority of refuges within the NWRS. In nearly half of the refuges, the onset of spring in recent decades is earlier than 95% of the historical range of spring onset dates since 1901. The spatial patterns and rates of advance in spring onset for refuges closely match those reported for the US National Park system [44], as well as previously documented regional- and national-scale patterns [32–34,45]. In addition, there is substantial spatial variation in response among refuges; for example, refuges in the southeastern US have exhibited little change, which is consistent with the “warming hole” previously reported for that region [34,62,63].

The potential consequences of advancing onset of spring within and across protected areas are wide-ranging; they include shifts in organismal sex ratios and decreased reproductive success in particular taxonomic groups [64,65], alterations in species abundance or distribution [66–68] through the development of temporal niche differentiation [69], the decoupling of existing phenological synchronies [70,71], and the production of more generations in an extended growing season [72,73]. Earlier onsets of spring can also affect the timing of activity of native or non-native nuisance species, including vectors of disease or invasive species [69,74]. Other more indirect effects on organisms or habitats may include impacts on seasonal or annual carbon storage and cycling [75], drought [45], or wildfire regimes [76]. The potential consequences for species of interest and early-season environmental conditions could undermine the mission of the refuge system or other national networks of protected areas; refuge units established expressly for the conservation of specific species may no longer be able to provide suitable habitat [77].

A clearer understanding of local to regional changes in the timing of spring onset can enhance seasonal planning and decision-making [78]. Given the priority that many refuge visitors place on bird watching [79], the effects of spring timing changes on resident and migratory bird populations may impact visitor services and management of visitation levels [80]. Researchers have linked climate change to increased rates of migratory vagrancy in some species [81]; the prospect of an exotic sighting could lead to occasional spikes in refuge visitation. Predicting popular seasonal events such as seasonal wildlife viewing or wildflower displays, or minimizing human activity within critical seasonal habitats, may become more difficult because of changes in spring conditions. Managers may need more up-to-date information on spring conditions, balanced with a more flexible and longer planning window for resource management planning, decision-making, and visitor management.

### Changes in spring onset within North American bird flyways over the last century

In all North American migratory flyways, spring is arriving earlier now than it has in the past. In three of the four flyways, the timing of spring has generally advanced more rapidly at higher latitudes, which is consistent with the observed trend of disproportionate warming with increasing latitudes across the Northern Hemisphere [36]. This pattern is apparent in the two mid-continent flyways but is most pronounced in the Atlantic flyway. Differential rates of spring advance within the Atlantic flyway may be attributable to the “warming hole” documented in the southeastern U.S. [34,63]; i.e., within the southern region of this flyway. This explanation may be applicable to the Mississippi and Central flyways, which also incorporate portions of the southeastern US.

Birds migrating along the Atlantic flyway–and to a lesser extent the Central and Mississippi flyways–may face unique challenges because of the disproportionate shifts in spring onset coupled with subsequent potential impacts on resources and habitats. Long-distance neotropical migrants crossing the Gulf of Mexico may arrive in the southern region of these flyways where onsets of spring do not exhibit a trend over the past several decades [82,83]. Refuges in this southeastern region of the US show relatively high variability in spring onset in recent years, suggesting that timing of spring onset is less consistent from year to year in this region than in others. This variability could cause issues for birds accustomed to arriving during coincident peaks in food resource availability. Further, a continued northward migration may present problems in habitat or resource availability; our results suggest that spring is advancing at a more rapid pace in the northern latitudes than in the southern latitudes of these flyways. Consequently, food resources may be past their peak when migrants arrive at breeding grounds, increasing the potential for trophic mismatches and attendant implications for population fitness [13,22,23,30,84–86].

Migratory birds utilizing the Pacific flyway may face different challenges. For example, if the birds are responding to exogenous migration cues, they may begin their northward migration lagging behind habitats and resources that have advanced in timing along the flyway. However, because the rate of advance in spring onset has not demonstrated significant trends across the latitudinal gradient of this flyway, the birds may be able to cue into their surrounding environment and adjust their rate of migration or routes to match local resource availability [29,37], or to match thermal environments as [38] recently observed within this flyway. Both short- and long-distance migrants using this flyway have shown an advance in the timing of spring migrations, though long-distance migrants have advanced to a lesser degree [87].

The timing and rate of migration in any given year can depend on a variety of cues, including atmospheric [26] and ecological conditions along the migratory route [27]. Considerable heterogeneity in terms of migratory arrival dates also exists within bird species [20,28]. Nonetheless, the ability of populations to maintain synchrony with the temporal windows of key seasonal phenomena may be critical for ensuring migratory success. Bird species that are unable to advance their overall migration timing have already suffered declines, while those with certain behavioral characteristics (e.g., longer migration distances) or specific habitat requirements may also be susceptible to mistimed arrivals relative to spring onset [12,14,15,20].

### Changes in spring onset within and between breeding and non-breeding ranges of two migratory bird species

Disproportionate advances in timing of spring onset–in FBI and to a lesser extent FLI–were observed for the breeding ranges of both examined bird species. Because FBI is most closely aligned with leaf-out in deciduous trees [48], these differential advances may affect the timing or abundance of insect food resources on which *V*. *cyanoptera* depends, requiring the birds to correspondingly adjust the timing or rate of migration to maintain synchrony (e.g., [13]). While this species has exhibited earlier arrivals at its breeding ground, the shift in migration timing still lags behind the shift in green-up timing [13]. Our analyses suggest that if *V*. *cyanoptera* continues to initiate migration north at a constant date, the birds are likely to find spring conditions similar to those of past years upon crossing the Gulf of Mexico. However, food resources may be past their peak in breeding ranges farther to the north, and the spatial homogeneity of FBI response across the majority of the breeding range may limit birds’ ability to make within-range distribution adjustments in response to unfavorable habitat conditions [37]. There is evidence of a northward shift to this species’ range [7], which may increase hybridization with the near-threatened golden-winged warbler (*Vermivora chrysoptera*) [88].

*G*. *americana* migrates entirely within the Central flyway, which generally exhibits a greater advance rate in the start of spring at higher vs. lower latitudes, consistent with trends in the birds’ breeding and non-breeding ranges. As with *V*. *cyanoptera*, *G*. *americana* could find food resources past peak upon arrival in the northern breeding ranges. The broad diet of *G*. *americana* [89] may mitigate the consequences of shifted resource availability for adult individuals during the breeding season. However, a static hatching phenology that is progressively decoupled from the earlier development of aquatic invertebrates and amphibians may reduce the availability of suitable prey for young birds [90].

The different extents of the two species’ seasonal habitats highlight the relevance of spatial domains for ensuring population resilience to ongoing and projected climate change. The constrained extent of *G*. *americana* breeding and non-breeding areas may be problematic if shifting seasonality patterns introduce detrimental effects on forage quality or ecosystem structure, such as the encroachment of woody vegetation into the preferred roosting habitat of floodplain meadows [91]. The birds’ preference for established territories, coupled with the widespread loss of suitable habitat due to agricultural conversions, preclude a spontaneous relocation to alternate breeding sites [90]. In contrast, the relatively broad extents of the *V*. *cyanoptera* breeding range allow for a more elastic response of individual birds to shift to within-range areas that may be more phenologically appropriate for breeding and food foraging requirements.

The earlier warming that underlies the advance of spring onset has the potential to indirectly alter critical habitat and food resource conditions through complex ecological dependencies. Although we have discussed some of the relevant links to spring onset (e.g., insect emergence), other secondary effects of seasonal alterations may be ultimately decisive for ensuring migratory population breeding success in the face of climate change.

### Considerations for resource management and additional research

For migratory species of special concern, USFWS could consider conservation and management across a variety of spatial scales, including refuges, seasonal ranges, and along-migration corridors that represent potentially critical transitional habitat [37]. The results of this study may help refuge managers formulate strategies for protecting these or other species likely to be affected by seasonal shifts. Potential management activities–many of which are already being employed—include eradication of invasive species, implementation of flooding or prescribed fire regimes (e.g., [90]), predator removal [92], and managed relocation to alternate sites [93]. At the refuge level, potential management actions include restoration and maintenance of habitat, such as planting species adapted to future climate conditions; assisted migration by translocating species; and providing additional food sources for early or late migrants. In some cases strategic acquisition of more suitable habitat may be necessary to accommodate changes for species of conservation concern [77].

A landscape-level approach may help elucidate co-varying impacts of changing spring onset on habitats and migrating species across full annual life cycles [94], requiring enhanced collaborations with conservation partners in adjacent lands [77,95]. For example, many neo-tropical migrants cross between flyways, following a clockwise, elliptical pattern that can take them into the Central, Mississippi, and Atlantic flyways [26]. Managers interested in evaluating impacts to these species may need to explore continental-scale patterns that transcend particular flyways, similar to efforts led by organizations such as Joint Ventures, the North American Bird Conservation Initiative, Partners in Flights, and others.

Future research could examine spatial variation in spring onset within each refuge, flyway, or seasonal habitat, or could consider phenological responses of plants or animals that affect habitat conditions or resource availabilities (i.e., the ecological processes and mechanisms that impact bird fitness). In addition, differences in bird species life-history or physiology may have important and potentially interactive effects on apparent phenological response to different driving variables [21,38]. As such, an important next step would be to integrate these results with geospatially controlled, *in-situ* ground-based bird data. Long-term phenological monitoring and new approaches and databases (e.g., eBird [96], which is the world’s largest biodiversity-related citizen science data collection effort) can help refuge managers understand the response of migrating birds and their habitats to earlier spring onset, and can facilitate strategies to inform decision-making processes [78]. The patterns in shifting spring onsets documented here may affect other guilds of migrating birds, such as hawks and shorebirds, as well as other migrating taxa including butterflies and ungulates [97]. The approach demonstrated in this study of evaluating spring advance across scales–from protected areas to seasonal habitats to migratory flyways–could be extended to other taxa that migrate on a seasonal basis.

## Conclusions

Our evaluation of changes in spring onset over the last century across the disparate spatial scales of the US wildlife refuge system, the seasonal ranges of two bird species, and the major North American flyways confirms that spring is arriving earlier in many areas of the continent. In fact, half of the refuges examined here are experiencing some of their earliest onset dates of spring–as measured by FLI and FBI–in recent decades relative to the last century. At the scale of seasonal migratory bird habitats, disproportionate rates of change over time have resulted in entire breeding ranges that have shifted to an earlier seasonality, whereas non-breeding ranges have remained relatively static. This pattern is also evident at the sub-continental scale of migratory flyways, where spring is advancing at a more rapid rate at higher latitudes than at lower latitudes in three of four flyways. The greatest differences in advance of spring onset were observed in the Atlantic flyway, which may be attributable to a “warming hole” documented within the southeastern US.

The substantial changes observed in the onset of spring across spatial scales present challenges for managers seeking to conserve and protect wildlife, plants, and habitats. An advancing spring has the potential to affect many facets of refuge management, including the timing of invasive and pest species detection and treatment, optimization of native seed collection, tracking seasonal distribution and abundance of disease vectors such as arthropod-borne viruses, monitoring and timing of management of wildlife, and visitor management. A more complete understanding of century-scale changes in habitats and seasonality across spatial scales should help resource managers make decisions that support effective conservation and management of natural resources under their purview.

## Supporting information

S1 Code (ZIP)

S1 Fig Change in the timing of spring onset for US National Wildlife Refuges. Changes are expressed in days per decade for First Leaf Index (FLI) and First Bloom Index (FBI) over the period 1901–2012.
(TIF)

S1 Table Spring onset metrics and trends for US National Wildlife Refuges. Area-weighted means; recent timing and variability; and temporal trends in First Leaf Index (FLI) and First Bloom Index (FBI) for each of the 496 analyzed refuges.
(XLSX)
