# Correction: Differential changes in the onset of spring across US National Wildlife Refuges and North American migratory bird flyways

**DOI:** 10.1371/journal.pone.0208348

**Published:** 2018-11-26

**Authors:** Eric K. Waller, Theresa M. Crimmins, Jessica J. Walker, Erin E. Posthumus, Jake F. Weltzin

In Fig 4 the labels for brown and red polygons are swapped in the map legend. Brown polygons should be the Early FLI & FBI and red polygons should be early FLI. Please see the corrected Fig 4 here.

**Fig 4:**
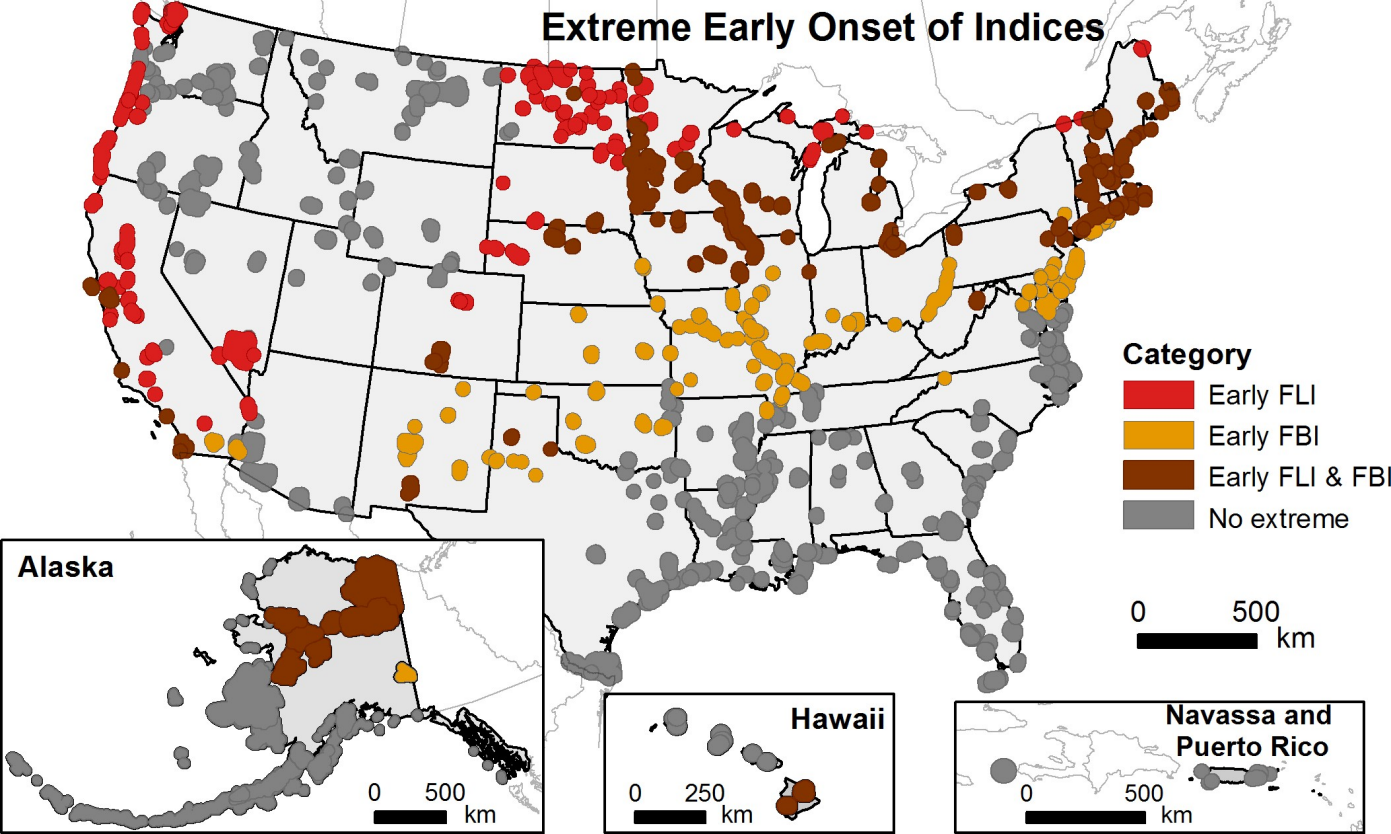
US National Wildlife Refuges with extremely early recent spring onset relative to the historical range of variability. Shown is the combination of selected data from Fig 3A and 3B; extremely early recent spring onset is defined as earlier than 95% of historical (1901–2012) First Leaf Index (FLI) or First Bloom Index (FBI) values.
