# Supplementary figures and images for: Differential changes in the onset of spring across US National Wildlife Refuges and North American migratory bird flyways
Source: PLoS One. 2018 Sep 12;13(9):e0202495. doi: 10.1371/journal.pone.0202495 (PMC6135358; doi:10.1371/journal.pone.0202495)

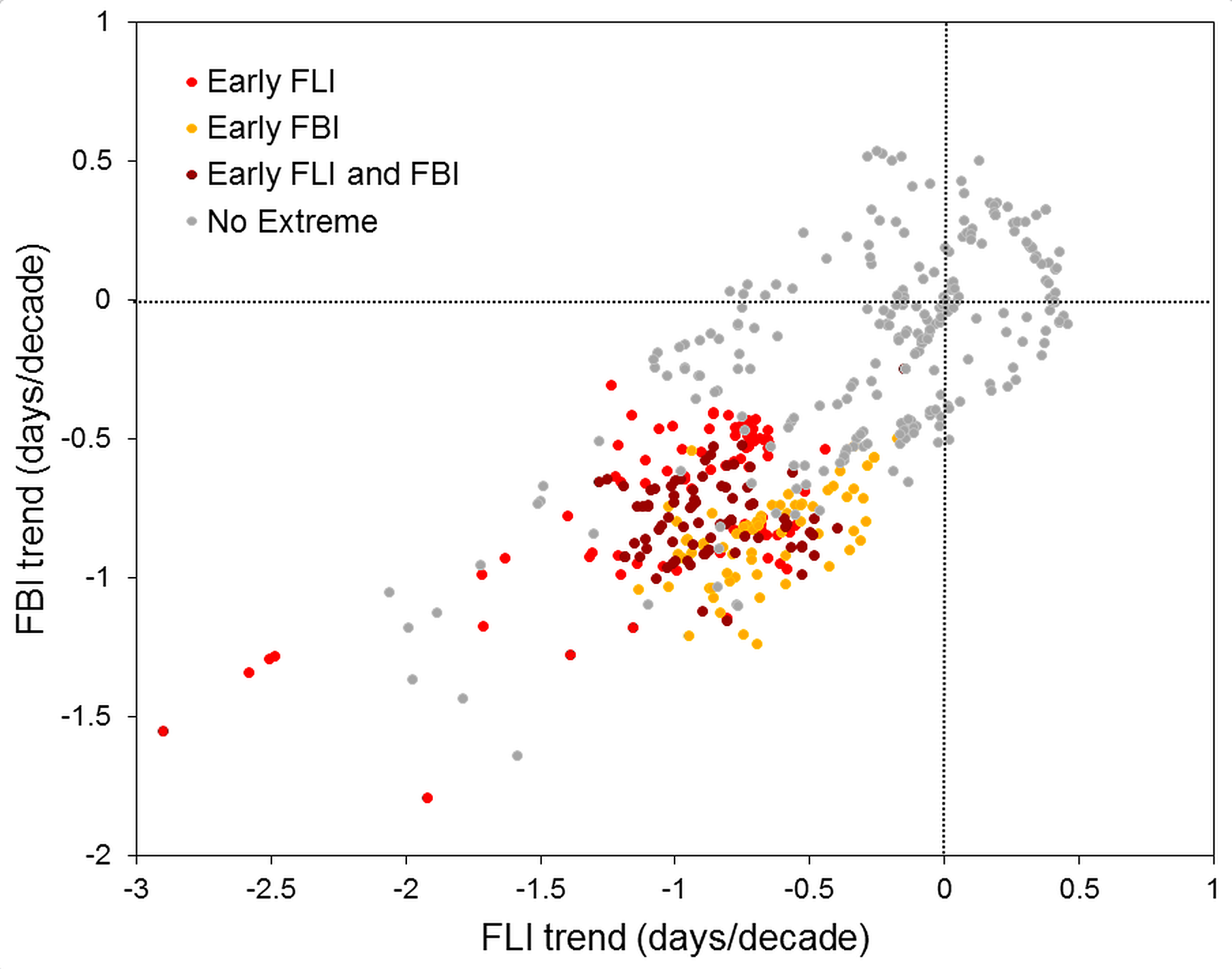

Supplement: S1 Fig — Changes are expressed in days per decade for First Leaf Index (FLI) and First Bloom Index (FBI) over the period 1901–2012. (TIF) [file pone.0202495.s002.tif]
